# AVE0991, a Nonpeptide Angiotensin 1-7 Receptor Agonist, Improves Glucose Metabolism in the Skeletal Muscle of Obese Zucker Rats: Possible Involvement of Prooxidant/Antioxidant Mechanisms

**DOI:** 10.1155/2020/6372935

**Published:** 2020-01-27

**Authors:** Viktoria Dobrocsyova, Miroslava Slamkova, Katarina Krskova, Lucia Balazova, Maciej Suski, Rafal Olszanecki, Sona Cacanyiova, Stefan Zorad

**Affiliations:** ^1^Institute of Experimental Endocrinology, Biomedical Centre, Slovak Academy of Sciences, Dúbravská cesta 9, 845 05 Bratislava 4, Slovakia; ^2^Chair of Pharmacology, Jagiellonian University Medical College, 31531 Krakow, Poland; ^3^Centre of Experimental Medicine, Slovak Academy of Sciences, Dúbravská cesta 9, 845 05 Bratislava 4, Slovakia

## Abstract

Angiotensin 1-7 (Ang 1-7) enhances insulin signaling and glucose transport activity in the skeletal muscle. The aim of our study was to evaluate the effect of AVE0991, a nonpeptide Mas receptor agonist, on the metabolic parameters, expression of RAS components and markers of oxidative stress, and insulin signaling in the skeletal morbidly obese rats. 33-week-old male obese Zucker rats were treated with vehicle and AVE0991 (0.5 mg/kg BW/day) via osmotic minipumps for two weeks. Gene expressions were determined by qPCR and/or Western blot analysis in musculus quadriceps. The enzymatic activities were detected flourometrically (aminopeptidase A) or by colorimetric assay kit (protein tyrosine phosphatase 1B). Administration of AVE0991 enhanced insulin signaling cascade in the skeletal muscle, reflected by improved whole-body glucose tolerance. It has been shown that reactive oxygen species (ROS) have insulin-mimetic action in muscle. The expression of renin receptor, transcription factor PLZF, and prooxidant genes was upregulated by AVE0991 accompanied by elevated expression of genes coding enzymes with antioxidant action. Our results show that AVE0991 administration activates genes involved in both ROS generation and clearance establishing a new prooxidant/antioxidant balance on a higher level, which might contribute to the improved insulin signaling pathway and glucose tolerance of obese Zucker rats.

## 1. Introduction

The renin-angiotensin system (RAS) is well known as an essential regulator of systemic blood pressure as well as fluid and electrolyte homeostasis. The classic understanding of the RAS has changed by the evidence of local tissue-specific formation of several RAS components, including skeletal muscle in vitro and in vivo. Local RAS in the skeletal muscle responds to physiological stimuli; it is capable of *de novo* angiotensin II (Ang II) production and can function independently of systemic RAS. Ang II has long been considered to be the single critical product of the RAS, which acts via two types of receptors: AT1 and AT2. Most of Ang II effects are mediated by the AT1 receptor, including vasoconstriction, hypertrophy, and cellular growth. Several lines of evidence have demonstrated the existence of two functionally opposing pathways of the RAS. The alternative pathway of the RAS involves the cleavage of Ang I or Ang II by angiotensin-converting enzyme 2 (ACE2) to Ang 1-7. Ang 1-7 acts via a Mas receptor and has antagonistic effects to the classical RAS pathway; it is able to improve insulin signaling and glucose transport activity in the skeletal muscle by enhanced insulin receptor substrate 1 (IRS1) and Akt kinase phosphorylation [1, 2]. Ang 1-7 has beneficial effect on skeletal muscle perfusion as well, since it evokes dilatation of precapillary arterioles. The major limitation of exogenous administration of Ang 1-7 is that it is a peptide with very short biological half-life and low oral bioavailability. AVE0991, a nonpeptide Mas receptor agonist, has been reported to mimic the effects of Ang 1-7. The main advantages of this compound are its stability, oral activity, and resistance against proteolytic enzymes [3, 4]. The effect of AVE0991 on the physiology of skeletal muscle has not been examined yet.

The aim of our study was to evaluate the effect of AVE0991 application on the (i) metabolic parameters, (ii) expression of the RAS components and (iii) markers of oxidative stress, and (iv) insulin signaling in the skeletal muscle of obese Zucker rats.

## 2. Materials and Methods

### 2.1. Animals

Male Zucker fatty rats (fa/fa) (*n* = 11) were purchased from Harlan (Udine, Italy). The animals were housed in a 12-hour light/dark cycle with access to water and standard diet ad libitum. Animals were separated to two groups. The control group was treated with vehicle (30% solution of cyclodextrin), and the experimental group received AVE0991 (Sanofi-Aventis, Frankfurt, Germany) (0.5 mg/kg BW/day in 30% solution of cyclodextrin) via osmotic minipumps (ALZET, CA, USA) for two weeks. The intraperitoneal glucose tolerance test (IPGTT) was performed to assess glucose clearance. On the 12th day, overnight-fasted animals were administered an i.p. injection of dextrose solution at a dose of 2 g/kg body weight. Glycaemia was measured in the tail vein blood immediately and in 30-minute intervals for 2 hours after glucose administration using a glucometer (Accu-Chek Active, Roche Diagnostics, Switzerland). After 2 days of recovery, overnight-fasted animals were sacrificed by decapitation at the age of 8 months. Experimental procedures involving animals were approved by the Jagiellonian University Ethical Committee on Animal Experiments.

### 2.2. Measurement of Selected Metabolic Parameters

Circulating insulin level was measured in plasma isolated from trunk blood samples obtained after decapitation using commercial radioimmunoassay kit (Millipore, Bedford, MA, USA) following the manufacturer's protocol. Lipid parameters were determined in the Laboratory Diagnostics Unit of the University Hospital in Krakow using commercially available kits (Roche Molecular Diagnostics, Pleasanton, CA, USA). Fasting glycaemia was analysed at SYNLAB (Bratislava, Slovakia) using multianalyzer COBAS INTEGRA 800 (Roche Diagnostics Ltd., Rotkreuz, Switzerland). Quantitative insulin sensitivity check index (QUICKI) was calculated as follows: inverse of the sum of the logarithms of the fasting insulin (*μ*U/ml) and fasting glucose (mg/dl).

### 2.3. RNA Isolation and Real-Time PCR

Prior to sampling for RNA, dissected samples of quadriceps muscle were snap-frozen and mashed under liquid nitrogen to powder. Samples were stored at -80°C until analyses. Total RNA was isolated from musculus quadriceps using RNeasy Plus Universal Mini Kit (Qiagen, Valencia, CA, USA), and reverse transcription was performed using Maxima First Strand cDNA Synthesis Kit (Thermo Fisher, Waltham, MA, USA) according to the manufacturer's protocol. Real-time PCRs were carried out applying Maxima SYBR Green qPCR Master Mix (Thermo Fisher, Waltham, MA, USA) and run on an ABI 7900HT thermal cycler (Applied Biosystems, Life Technologies, Carlsbad, CA, USA) using rat-specific primer pairs shown in Table 1. Data were normalized to the expression of housekeeping gene ribosomal protein S29 (Rps29) which was not altered by the treatment.

### 2.4. Western Blotting

Samples of musculus quadriceps were homogenized using a glass Teflon homogenizer in an ice-cold lysis buffer (10 mM Tris-HCl, pH 8.0, 150 mM NaCl, 1% Nonidet P-40, 0.5% sodium deoxycholate, 0.1% SDS, 0.5 mM dithiothreitol, 1 mM phenylmethylsulphonyl fluoride, 5 mg/ml leupeptin, and 5 mg/ml aprotinin). Homogenates were placed on ice for 2 h with occasional mixing and centrifuged at 16000 g/20 min/4°C. The supernatant was used for Western blot analysis. Protein concentration was determined by Bicinchoninic Acid Protein Assay Kit (Sigma-Aldrich, St. Louis, MO, USA) according to the manufacturer's instructions. Proteins were separated by SDS-PAGE technique on 10% polyacrylamide gels and electrotransferred in semidry conditions to a low-fluorescence PVDF membrane (Immobilon-FL, Millipore, Bedford, MA, USA). The equal loading and transfer was confirmed by Ponceau staining (SERVA, Heidelberg, Germany). Membranes were blocked in 5% bovine serum albumin (BSA) in Tris-Buffered Saline for 1 h at room temperature and incubated overnight at 4°C with primary antibody against insulin receptor *β* (#3025, Abcam, Cambridge, MA, USA) diluted 1 : 1000, phospho-IGF-I receptor *β* (Tyr1135/1136)/insulin receptor *β* (Tyr1150/1151) (#3024, Abcam, Cambridge, MA, USA) diluted 1 : 1000, IRS1 (#2382, Abcam, Cambridge, MA, USA) diluted 1 : 1000, phospho-IRS1 (Tyr896) (LS-C381052, LifeSpan BioSciences, Inc., Seattle, WA, USA) diluted 1 : 500, phospho-IRS1 (Ser307) (#2381, Abcam, Cambridge, MA, USA) diluted 1 : 1000, phospho-IRS1 (Ser612) (#2386, Abcam, Cambridge, MA, USA) diluted 1 : 1000, Akt (#9272, Abcam, Cambridge, MA, USA) diluted 1 : 1000, phospho-Akt (Thr308) (#9275, Abcam, Cambridge, MA, USA) diluted 1 : 1000, PTP1B (LS-C385670, LifeSpan BioSciences, Inc., Seattle, WA, USA) diluted 1 : 1500, and phospho-PTP1B (Ser50) (LS-C381317, LifeSpan BioSciences, Inc., Seattle, WA, USA) diluted 1 : 1500 in 5% BSA in Tris-Buffered Saline. After washing in TBS with Igepal, the membranes were incubated with fluorescently labelled secondary anti-rabbit (#5151) or anti-mouse (#5257) antibodies (Cell Signaling Technology, Danvers, MA, USA) diluted 1 : 15000 for 1 h at room temperature. Infrared fluorescence was detected using the Odyssey Infrared Imaging System (LI-COR Biosciences, Lincoln, NE, USA), and Odyssey IR Imaging System software version 2.0 was used for analysis. As the protein expressions of the tested endogenous loading controls (GAPDH, *β*-actin, and *α*-tubulin) displayed significant variations upon obesity [5] and AVE0991 treatment; the signal intensities of the proteins of interest were normalized to the sample's total protein content stained with Coomassie Brilliant Blue [6]. Briefly, after membrane scanning and subsequent washing, blots were stained for 1 min (0.04% Coomassie Brilliant Blue (*w*/*v*), 40% methanol (*v*/*v*), and 5% acetic acid (*v*/*v*)), destained for 2 min (40% methanol (*v*/*v*) and 5% acetic acid (*v*/*v*)), washed in water, and dried. Signal corresponding to total proteins on the blot was analysed by software ImageJ 1.42q (NIH, USA).

### 2.5. Measurement of Enzyme Activities

Skeletal muscle was homogenized using a glass Teflon homogenizer in lysis buffer (250 mM saccharose, 10 mM Tris, and pH 7.4). The homogenate was centrifuged at 1000 x g/10 min/4°C. The supernatant was collected and centrifuged at 16000 x g/15 min/4°C to separate the membrane fraction. Protein concentration was measured by the Bicinchoninic Acid Protein Assay (Sigma-Aldrich, St. Louis, MO, USA). Activity of aminopeptidase A (APA) was determined in the membrane fraction. The samples were mixed with substrate solution containing 10 mg/100 ml bovine serum albumin, 10 mg/100 ml dithiothreitol, 50 mM CaCl2 in 50 mM Tris pH 7.4, and 100 mM H-Glu-*β*-naphthylamide (Bachem, Bubendorf, Switzerland), which served as a substrate for APA. The 96-well was placed in a Synergy™ H4 Hybrid Reader (BioTek, Winooski, VT, USA) fluorimeter and preheated to 37°C. The enzyme kinetics was measured during 60 minutes in 5-minute intervals as the amount of *β*-naphthylamide was released from the substrate due to the enzyme activity of APA at wavelengths 340 nm (excitation) and 410 nm (emission). Enzyme activity is expressed in micromoles per liter of H-Glu-*β*-naphthylamide hydrolysed per minute per milligram of protein. Activity of PTP1B was determined in the cytosolic fraction using the commercially available PTP1B Assay Kit, Colorimetric (539736, Merck Millipore, Burlington, MA, USA), following the manufacturer's protocol.

### 2.6. Statistical Analysis

The results are presented as mean ± S.E.M. Analysis of normally distributed data was performed using the Kolmogorov-Smirnov test. Nonnormally distributed data were subjected to natural logarithm transformation prior to statistical analysis. Differences between experimental groups were analysed by Student's *t*-test. Overall level of statistical significance was reached at ^∗^*p* < 0.05, ^∗∗^*p* < 0.01, and ^∗∗∗^*p* < 0.001.

## 3. Results and Discussion

**Figure 1:**
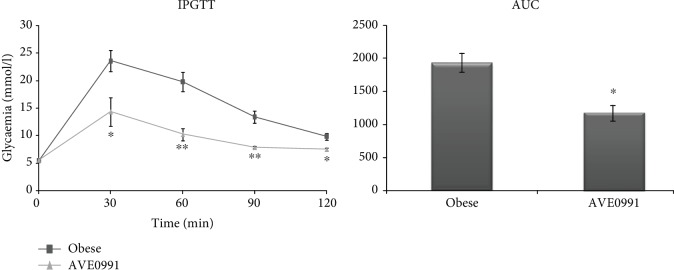
Intraperitoneal glucose tolerance test (IPGTT) evaluated in control obese Zucker rats (*n* = 6) and in obese Zucker rats receiving AVE0991 (*n* = 5) via osmotic minipumps for two weeks. AUC: area under curve. Data are presented as mean ± S.E.M. and were analysed using Student's *t*-test, ^∗^*p* < 0.05; ^∗∗^*p* < 0.01.

Recessively homozygous (fa/fa) Zucker rats display obesity, hyperleptinaemia, hyperinsulinaemia, hypercholesterolaemia, and hypertriglyceridaemia [7]. Body weight and metabolic parameters observed in plasma were not significantly changed after AVE0991 administration for two weeks (Table 2). On the other hand, AVE0991 administration significantly improved glucose tolerance evaluated by IPGTT (Figure 1).

### 3.1. Local RAS Components

**Figure 2:**
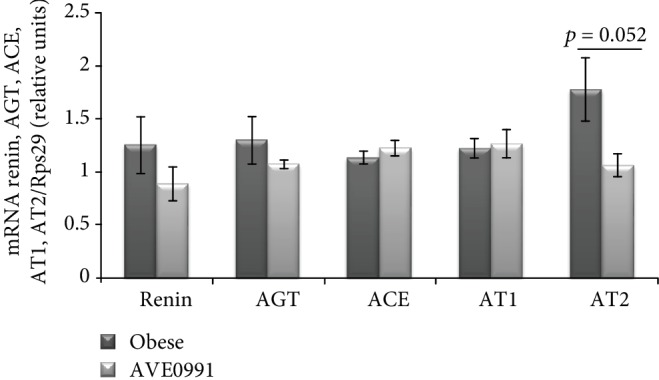
Classical RAS pathway components in the skeletal muscle of obese Zucker rats. Gene expression of renin, angiotensinogen (AGT), angiotensin-converting enzyme (ACE), angiotensin II type 1 receptor (AT1), and angiotensin II type 2 receptor (AT2) in musculus quadriceps of control obese Zucker rats and in obese Zucker rats receiving AVE0991 via osmotic minipumps for two weeks, determined by real-time PCR. Data were normalized to the gene expression of 40S ribosomal protein S29 (Rps29) whose expression was not altered by the treatment. Data presented as mean ± S.E.M. were analysed by Student's *t*-test.

The expression of the classical RAS components, namely, renin, angiotensinogen (AGT), ACE, AT1, and AT2, was not changed in the skeletal muscle of obese Zucker rats upon AVE0991 treatment. However, the expression of AT2 tended (*p* = 0.052) to decrease after AVE0991 administration (Figure 2).

**Figure 3:**
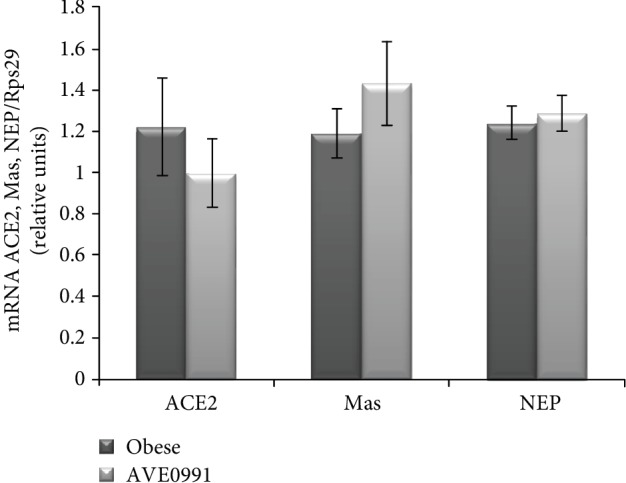
Alternative RAS pathway components in the skeletal muscle of obese Zucker rats. Gene expression of angiotensin-converting enzyme2 (ACE2), Mas receptor, and neutral endopeptidase (NEP) in musculus quadriceps of control obese Zucker rats and in obese Zucker rats receiving AVE0991 via osmotic minipumps for two weeks, determined by real-time PCR. Data were normalized to the gene expression of 40S ribosomal protein S29 (Rps29) whose expression was not altered by the treatment. Data presented as mean ± S.E.M. were analysed by Student's *t*-test.

Similarly, the expression of the alternative RAS components ACE2, Mas, and neutral endopeptidase (NEP) was unchanged in the skeletal muscle of obese Zucker rats after AVE0991 application for two weeks (Figure 3).

**Figure 4:**
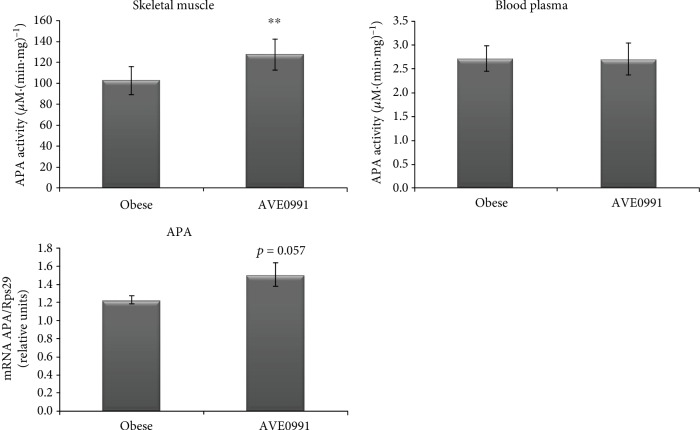
Plasma and skeletal muscle APA activity. Enzyme activity of aminopeptidase A (APA) measured in the membrane fraction isolated from the skeletal muscle and the blood plasma control obese Zucker rats and in obese Zucker rats receiving AVE0991 via osmotic minipumps for two weeks. Gene expression of aminopeptidase A (APA) in musculus quadriceps determined by real-time PCR. Data were normalized to the gene expression of 40S ribosomal protein S29 (Rps29) whose expression was not altered by the treatment. Data presented as mean ± S.E.M. were analysed by Student's *t*-test, ^∗^*p* < 0.05; ^∗∗^*p* < 0.01; ^∗∗∗^*p* < 0.001.

The expression of APA in the skeletal muscle tended to increase (*p* = 0.057) by AVE0991 administration. Correspondingly, the activity of APA was significantly elevated (*p* < 0.01) in the skeletal muscle of obese Zucker rats treated with AVE0991. The plasma APA activity was not affected by the treatment (Figure 4).

**Figure 5:**
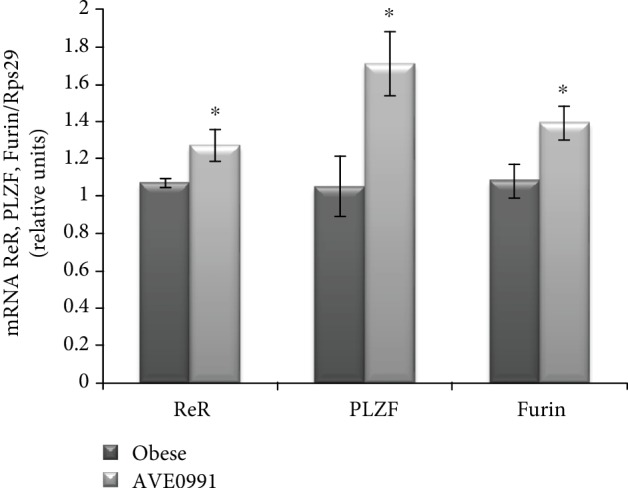
Renin receptor in the skeletal muscle of Zucker rats. Gene expression of the renin receptor (ReR) at the level of mRNA (a) and protein with representative Western blots (b); mRNA expression of the transcription factor promyelocytic leukemia zinc finger (PLZF, Zbtb16) and furin in musculus quadriceps of control obese Zucker rats and in obese Zucker rats receiving AVE0991 via osmotic minipumps for two weeks. Levels of mRNA were determined by real-time PCR. Data were normalized to the gene expression of 40S ribosomal protein S29 (Rps29) whose expression was not altered by the treatment. The amount of renin receptor protein was quantified by Western blot method. The signal intensities of the proteins of interest were normalized to the sample's total protein content stained with Coomassie Brilliant Blue. Data are presented as mean ± S.E.M. Results were analysed by Student's *t*-test, ^∗^*p* < 0.05.

Statistical analyses revealed a significant (*p* < 0.05) increase of renin receptor (ReR) expression in the skeletal muscle of obese Zucker rats receiving AVE0991 when compared to the control group. The mRNA level of promyelocytic leukemia zinc finger (PLZF; Zbtb16), the repressor of ReR transcription, was examined as well. We observed a significant upregulation of PLZF gene expression (*p* < 0.05) induced by AVE0991 treatment (Figure 5).

### 3.2. Markers of Oxidative Stress and Antioxidant Defence Mechanism

**Figure 6:**
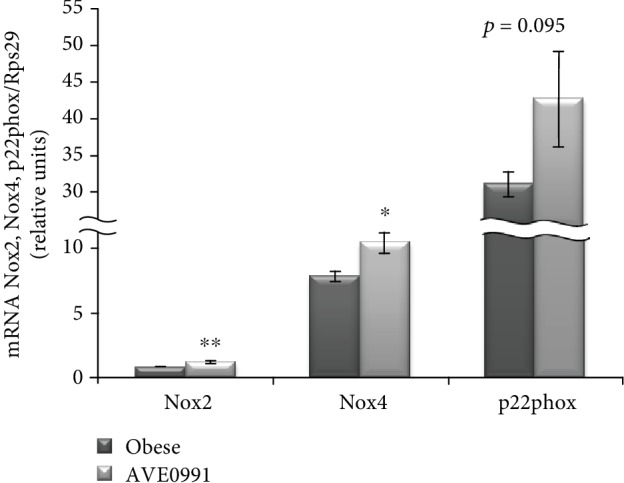
Prooxidant genes in the skeletal muscle of obese Zucker rats. Gene expression of NADPH oxidase 2 (Nox2), Nox4, and p22phox in musculus quadriceps of control obese Zucker rats and in obese Zucker rats receiving AVE0991 via osmotic minipumps for two weeks, determined by real-time PCR. Data were normalized to the gene expression of 40S ribosomal protein S29 (Rps29) whose expression was not altered by the treatment. Data presented as mean ± S.E.M. were analysed by Student's *t*-test, ^∗^*p* < 0.05; ^∗∗^*p* < 0.01.

Since ReR activation is involved in the regulation of NADPH oxidase 2 and 4 (Nox2 and Nox4) expression, we evaluated the mRNA levels of Nox2, Nox4, and p22phox, the *α*-subunit of NADPH oxidase system. The expression of Nox2 and Nox4 was elevated significantly (*p* < 0.05), while the expression of p22phox was not affected significantly (*p* = 0.095) by AVE0991 administration in the skeletal muscle of obese Zucker rats (Figure 6).

**Figure 7:**
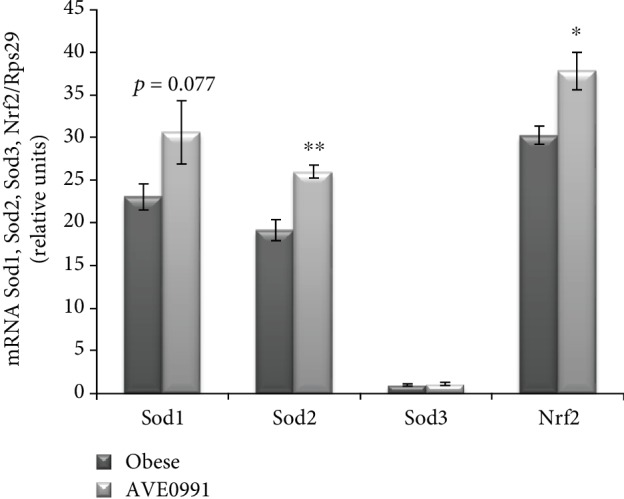
Genes coding enzymes with antioxidant action in the skeletal muscle of obese Zucker rats. Gene expression of superoxide dismutase 1 (Sod1), Sod2, Sod3, and nuclear factor erythroid 2-related factor 2 (Nrf2) in musculus quadriceps of control obese Zucker rats and in obese Zucker rats receiving AVE0991 via osmotic minipumps for two weeks, determined by real-time PCR. Data were normalized to the gene expression of 40S ribosomal protein S29 (Rps29) whose expression was not altered by the treatment. Data presented as mean ± S.E.M. were analysed by Student's *t*-test, ^∗^*p* < 0.05; ^∗∗^*p* < 0.01.

The expression of enzymes with antioxidant properties was affected by AVE0991 treatment as well. Superoxide dismutase 2 (Sod2) (*p* < 0.05) and nuclear factor erythroid 2-related factor 2 (Nrf2) (*p* < 0.01) mRNAs were significantly enhanced in the muscle of the treated group when compared to the control group. The expression of Sod1 and Sod3 was not affected significantly by the treatment (Figure 7).

### 3.3. Insulin Signaling

**Figure 8:**
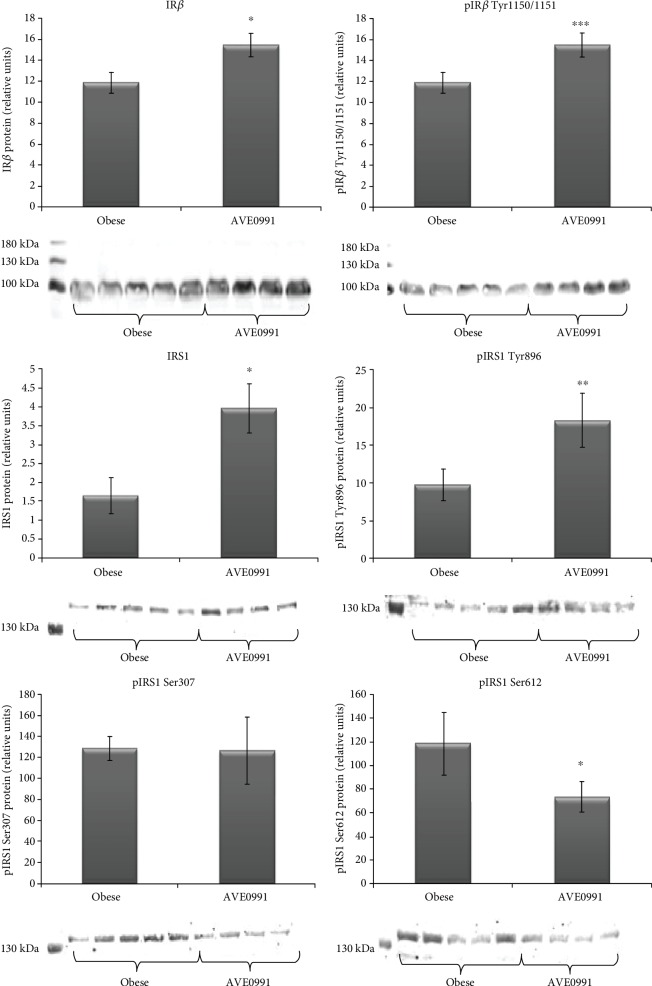
Insulin signaling cascade in the skeletal muscle of obese Zucker rats. Protein content of total insulin receptor *β* subunit (IR*β*), phosphorylated IR*β* at Tyr1150/1151, total insulin receptor substrate 1 (IRS1), and phosphorylated IRS1 at Tyr896, Ser307, and Ser612 with representative Western blots in musculus quadriceps of control obese Zucker rats and in obese Zucker rats receiving AVE0991 via osmotic minipumps for two weeks. The amount of protein was quantified by Western blot method. The signal intensities of the proteins of interest were normalized to the sample's total protein content stained with Coomassie Brilliant Blue. Data are presented as mean ± S.E.M. Results were analysed by Student's *t*-test, ^∗^*p* < 0.05; ^∗∗^*p* < 0.01; ^∗∗∗^*p* < 0.001.

Regarding the insulin signaling cascade, the protein expression and phosphorylation of insulin receptor *β* subunit (IR) at Tyr1150/1151 were both significantly (*p* < 0.05 and *p* < 0.001) upregulated in the skeletal muscle of obese Zucker rats receiving AVE0991. The protein content of total insulin receptor substrate (IRS1) was significantly (*p* < 0.05) increased in the treated group. We evaluated the phosphorylation of IRS1 at different amino acid residues. Upon AVE0991 administration, IRS1 phosphorylation on Tyr896 was increased significantly (*p* < 0.01), while Ser612 phosphorylation was decreased (*p* < 0.05). The protein content of phosphorylated IRS1 on Ser307 was unchanged by the treatment (Figure 8).

The ratio of pIR*β* at Tyr1150/1151 and total protein content of IR*β*, respectively, and the ratio of pIRS1 on various amino acid residues and total protein content of IRS1 were calculated (Table 3). The ratios of pIR*β* (Tyr1150/1151)/IR*β*, pIRS1 (Tyr896)/IRS1, and pIRS1 (Ser307)/IRS1 were unaffected by the treatment, while pIRS1 (Ser612)/IRS1 ratio was significantly reduced by AVE0991 application.

**Figure 9:**
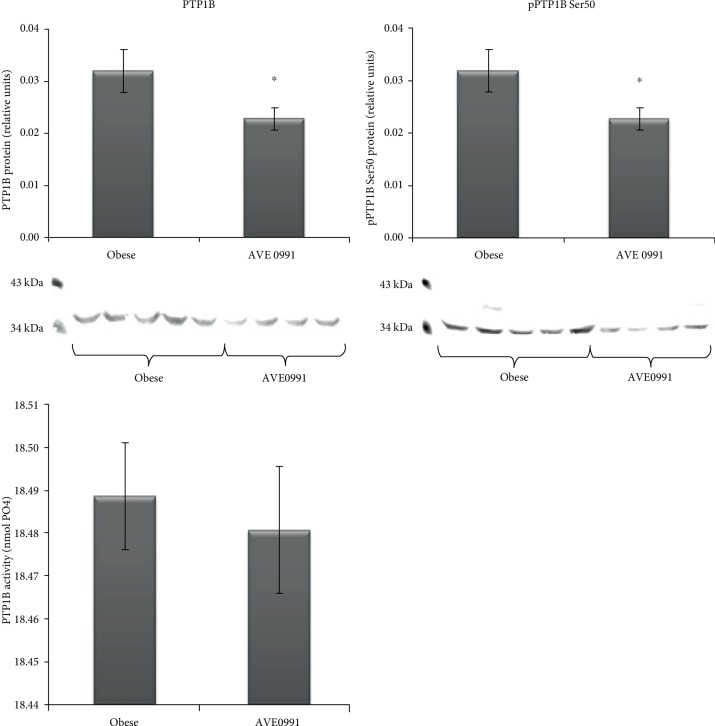
Protein tyrosine phosphatase 1B (PTP1B) in the skeletal muscle of obese Zucker rats. Protein content of total protein tyrosine phosphatase 1B (PTP1B), phosphorylated PTP1B at Ser50 with representative Western blots, and PTP1B activity in musculus quadriceps of control obese Zucker rats and in obese Zucker rats receiving AVE0991 via osmotic minipumps for two weeks. The amount of protein was quantified by Western blot method, and PTP1B activity was measured using commercially available colorimetric assay kit. The signal intensities of the proteins of interest were normalized to the sample's total protein content stained with Coomassie Brilliant Blue. Data are presented as mean ± S.E.M. Results were analysed by Student's *t*-test, ^∗^*p* < 0.05; ^∗∗^*p* < 0.01; ^∗∗∗^*p* < 0.001.

The protein content of protein tyrosine phosphatase 1B (PTP1B), a negative regulator of the insulin signaling cascade, was (*p* < 0.05) decreased, accompanied by reduced PTP1B Ser50 phosphorylation (*p* < 0.05) in the skeletal muscle of obese Zucker rats receiving AVE0991. However, the activity of PTP1B in the skeletal muscle was unaffected by the treatment (Figure 9).

## 4. Discussion

Obese Zucker rats represent a well-known model of the prediabetic state characterized by peripheral insulin resistance, which is caused by an impaired insulin-stimulated glucose uptake in skeletal muscle [8]. Moreover, these rats display dyslipidemia, hyperinsulinemia [9], and a reduced microvessel density in the musculature [10]. Several lines of evidence show that the alternative ACE2/Ang 1-7/Mas pathway has positive metabolic effects in the skeletal muscle [1, 2]. Since the application of the peptide Ang 1-7 is limited by its chemical properties, the nonpeptide Mas receptor agonist—AVE0991—represents a promising compound in pharmacological augmentation of the ACE2/Ang 1-7/Mas axis [3, 4]. In our study, obese Zucker rats were administered AVE0991 for two weeks via osmotic minipumps. The metabolic parameters observed in the plasma were unchanged after the treatment. However, the glucose tolerance evaluated by IPGTT was significantly improved by AVE0991.

Since AVE0991 acts as an activator of the alternative RAS pathway, and it is known that local skeletal muscle RAS is affected by obesity, we evaluated the application of AVE0991 on the expression of the local RAS components in the skeletal muscle of obese Zucker rats. However, we did not find any significant alterations in the expression pattern of the classical (AGT, ACE, AT1, and renin) and/or the alternative RAS pathway components (ACE2, Mas, and NEP) by the treatment. In our previous study, we examined the effect of obesity on the expression of RAS components in the skeletal muscle. We detected a significant obesity- and impaired glucose tolerance-related increase of AT2 expression in the group of 8-month-old Zucker rats [5]. On the contrary, AVE0991 normalized AT2 expression in muscle of age-matched animals (present study), indicating that impaired AT2 expression is partially reversed by AVE0991 administration.

The membrane-bound enzyme, aminopeptidase A (glutamyl aminopeptidase), catalyses the cleavage of Ang II to Ang III [11, 12]. The origin of the soluble form in the plasma has not been clarified yet, since there is no evidence for active secretion from any of the tissues. We have recently detected a significant decrease of membrane-bound APA activity in the skeletal muscle of Zucker rats by obesity, which negatively correlated with the plasma APA activity [5]. Simultaneously, the plasma cholesterol levels significantly correlated with the plasma APA activity. These data suggest that elevated plasma cholesterol in obesity might have a stimulatory effect on membrane-bound APA autolysis in the skeletal muscle, which in turn contributes to the rise of plasma APA activity in obese individuals. After AVE0991 treatment, we detected a significant increase of membrane-bound APA activity accompanied by an increasing tendency of APA expression in the skeletal muscle, which opposes the obesity-induced decrease of muscle APA activity described earlier [5]. It implies that AVE0991 has the potential to normalise skeletal muscle membrane-bound APA activity and expression in obese Zucker rats.

Recent studies suggest the existence of a novel pathway, where renin can act in a hormone-like fashion via the renin receptor. The catalytic activity of renin is increased 4- to 5-fold when bound to its receptor. Moreover, prorenin which was considered an inactive precursor of renin gains enzymatic activity without proteolytical cleavage of the prosegment after binding to the ReR [13]. It is known that ReR mediates the production of ROS by both Ang II-dependent and Ang II-independent pathways and regulates the expression of the Nox family members [14, 15]. Here, we show for the first time that AVE0991 treatment elevates expression of ReR in the skeletal muscle. Furthermore, the expression of Nox2 and Nox4 is elevated as well by AVE0991 application; however, this elevation might occur via other mechanisms, which have not been elucidated yet.

To the best of our knowledge, this is the first study showing the divergent action of AVE0991. On the one hand, it elevates the expression of prooxidative genes (Nox2, Nox4), while on the other hand, the expression of genes with antioxidative properties (Sods, Nrf2) is elevated upon AVE0991 treatment, establishing a new pro- and antioxidant equilibrium on a higher level. Several lines of evidence support a pivotal role of endogenous ROS in the insulin signaling cascade and subsequent glucose uptake by the skeletal muscle [16–19]. Moreover, the application of antioxidants has a negative effect on skeletal muscle glucose utilisation after exercise [17]. It has been proven that Nox4 inactivation results in inhibition of insulin signaling cascade, which means that for proper signal transduction, a certain amount of ROS is needed [20]. Insulin after binding to its receptor triggers rapid generation of ROS via activation of Nox4 in insulin-responsive tissues, which facilitates signal transduction through the insulin action pathway [20–22]. This mechanism is mediated by the oxidative inhibition of protein tyrosine phosphatase 1B (PTP1B) by ROS. The molecule of PTP1B has a catalytic activity if its thiol moiety is in a reduced form, when it acts inhibitory onto insulin signal transduction. The IR*β* subunit domain must be autophosphorylated to achieve full receptor kinase activation, which is a target of several protein phosphatase regulators of insulin signaling, including PTP1B [23]. By these means, the upregulated expression of ReR and Nox4 might contribute to oxidative inhibition of PTP1B and subsequent facilitation of insulin signal transduction. In order to confirm this hypothesis, we evaluated the insulin signal transduction and PTP1B protein expression and activity in the skeletal muscle of obese Zucker rats after AVE0991 treatment. We found a significant decrease of PTP1B protein content as well as decreased PTP1B Ser50 phosphorylation. However, these changes were not reflected in protein PTP1B activity measured by colorimetric assay. It should be noted that in vitro conditions are comprising optimal amount of the phosphopeptide substrate in the reaction, which might not be the case in in vivo conditions. Despite unchanged PTP1B activity detected, we found beneficial changes in the insulin signaling cascade. Total IR*β* protein content was increased in treated animals. In addition to increased tyrosine phosphorylation of the IR*β* subunit, the experimental group displayed a significant increase in both total IRS1 and pIRS1 Tyr896. Since both the total protein and the phosphorylated forms were elevated, the ratios of pIR*β*/IR*β* and pIRS1/IRS1 at tyrosine amino acid residues were unaffected. On the other hand, the protein content of phosphorylated IRS1 at Ser612 and the ratio of pIRS1 (Ser612)/IRS1 were significantly decreased. These positive changes show that AVE0991 treatment has a beneficial effect on the insulin signaling cascade in the skeletal muscle, which is also reflected by the improvement of glucose tolerance in treated animals. Our results indicate that the improvement of insulin signaling cascade occurred via inhibition of IRS1 serine phosphorylation rather than by increased tyrosine kinase activity in the skeletal muscle.

## 5. Conclusions

In summary, our results show for the first time that the nonpeptide Mas agonist—AVE0991—improved the obesity-induced metabolic alterations in the skeletal muscle, which was reflected by improvement of the whole-body glucose tolerance accompanied by elevated IR*β* and IRS total protein content and tyrosine phosphorylation and decreased serine phosphorylation. The positive effects of AVE0991 on skeletal muscle glucose utilisation might be—at least partially—explained by the activation of genes involved in ROS generation and inhibition of PTP1B protein content. This mechanism is accompanied by activation of antioxidant mechanisms, and therefore, a new prooxidant/antioxidant balance is established on a higher level. These findings open a new possibility for the treatment of obesity-induced metabolic alterations in the skeletal muscle based on mimicking the endogenous effects of Ang 1-7.

## Figures and Tables

**Table 1:** Primer sequences used for qPCR.

*Ace*	Fw	5′-ATG GTA CAG AAG AAG GGC TGG AA-3′
	Rv	5′-TTG TAG AAG TCC CAC GCA GA-3′

*Ace2*	Fw	5′-TCA GAG CTG GGA TGC AGA AA-3′
	Rv	5′-GGC TCA GTC AGC ATG GAG TTT-3′

*Agt*	Fw	5′-CAT GAG TTC TGG GTG GAC AA-3′
	Rv	5′-AAG TTG TTC TGG GCG TCA CT-3′

*Apa* (*Enpep*)	Fw	5′-GGC TCC CTT GTG GGT TTT TAC-3′
	Rv	5′-TCT TGT TGG GTT CAT CGA AAC A-3′

*AT_1_* (*Agtr1a*)	Fw	5′-TCT CAG CAT CGA TCG CTA CCT-3′
	Rv	5′-AGG CGA GAC TTC ATT GGG TG-3′

*AT_2_*	Fw	5′-ACC TTT TGA ACA TGG TGC TTT G-3′
	Rv	5′-GTT TCT CTG GGT CTG TTT GCT C-3′

*Furin*	Fw	5′-GGG TTT CCC AGC AGT CTT CA-3′
	Rv	5′-GCC AGA TCC CCA GGT GTG-3′

*Mas1* (*MasR*)	Fw	5′-TGA CCA TTG AAC AGA TTG CCA-3′
	Rv	5′-TGT AGT TTG TGA CGG CTG GTG-3′

*Nep* (*Mme*)	Fw	5′-GCA GAA ATC AGA TCG TCT TCC CCG-3′
	Rv	5′-CTG AGT CCA CCA GTC AAC GAG GT-3′

*Nox2*	Fw	5′-TGA TCA TCA CAT CCT CCA CCA A-3′
	Rv	5′-GAT GGC AAG GCC GAT GAA-3′

*Nox4*	Fw	5′-CTGCATCTGTCCTGAACCTCAA-3′
	Rv	5′-TCTCCTGCTAGGGACCTTCTGT-3′

*Nrf2*	Fw	5′-GTT GAG AGC TCA GTC TTC AC-3′
	Rv	5′-CAG AGA GCT ATC GAG TGA CT-3′

*p22^phox^*	Fw	5′-TGGCCTGATCCTCATCACAG-3′
	Rv	5′-AGGCACGGACAGCAGTAAGT-3′

*Plzf* (*Zbtb16*)	Fw	5′-GCGAAGAAGAAGAGGACCGTAAG-3′
	Rv	5′-CCGGAATGCTTCGAGATGAA-3′

*Renín*	Fw	5′-CCA CCT TCA TCC GCA AGT TC-3′
	Rv	5′-TGC GAT TGT TAT GCC GGT C-3′

*Rer* (*Atp6ap2*)	Fw	5′-TGG CCT ATA CCA GGA GAT CG-3′
	Rv	5′-AAT AGG TTG CCC ACA GCA AG-3′

*Rps29*	Fw	5′-GCTGAACATGTGCCGACACT-3′
	Rv	5′-GGTCGCTTAGTCCAACTTAATGAA-3′

*Sod1*	Fw	5′-CAC TCT AAG AAA CAT GGC G-3′
	Rv	5′-CTG AGA GTG AGA TCA CAC G-3′

*Sod2*	Fw	5′-TTC AGC CTG CAC TGA AG-3′
	Rv	5′-GTC ACG CTT GAT AGC CTC-3′

*Sod3*	Fw	5′-CTT GAC CTG GTT GAG AAG ATA G-3′
	Rv	5′-GAT CTG TGG CTG ATC GG-3′

**Table 2:** Selected metabolic parameters of control obese Zucker rats and in obese Zucker rats receiving AVE0991 via osmotic minipumps for two weeks.

	Obese control	AVE0091	Statistics
	*n* = 5	*n* = 4	
Body weight (g)	613.00 ± 3.64	621.10 ± 4.29	*p* = 0.877
Fasting glycaemia (mmol/l)	6.42 ± 0.10	6.15 ± 0.22	*p* = 0.261
Insulin (ng/ml)	10.28 ± 1.06	10.14 ± 0.32	*p* = 0.914
QUICKI^1^	0.221 ± 0.002	0.222 ± 0.001	*p* = 0.829
Triglycerides (mmol/l)	3.59 ± 0.69	5.16 ± 1.05	*p* = 0.236
Cholesterol (mmol/l)	6.01 ± 0.35	7.13 ± 0.82	*p* = 0.254
HDL (mmol/l)	2.39 ± 0.25	2.00 ± 0.48	*p* = 0.462
LDL (mmol/l)	0.95 ± 0.04	1.36 ± 0.37	*p* = 0.196
VLDL (mmol/l)	1.63 ± 0.32	2.35 ± 0.48	*p* = 0.236
LDL/HDL	0.42 ± 0.05	0.66 ± 0.14	*p* = 0.110

^1^QUICKI: quantitative insulin sensitivity check index.

**Table 3:** Insulin signaling cascade in the skeletal muscle of control obese Zucker rats and in obese Zucker rats receiving AVE0991 via osmotic minipumps for two weeks.

Ratio	Obese control	AVE0091	Statistics
	*n* = 5	*n* = 4	
pIR*β* (Tyr1150/1151)/IR*β*	0.29 ± 0.02	0.27 ± 0.01	*p* = 0.689
pIRS1 (Tyr896)/IRS1	5.23 ± 1.42	4.94 ± 2.14	*p* = 0.884
pIRS1 (Ser307)/IRS1	66.69 ± 10.94	36.57 ± 13.62	*p* = 0.151
pIRS1 (Ser612)/IRS1	50.02 ± 9.93	21.39 ± 2.57	*p* < 0.05

Insulin receptor *β* subunit (IR*β*), phosphorylated IR*β* at Tyr1150/1151, total insulin receptor substrate 1 (IRS1), and phosphorylated IRS1 at Tyr896, Ser307 and Ser612 residues.

## Data Availability

The data used to support the findings of this study are available from the corresponding author upon request.
